# Trends in suicide in Scotland 1981 – 1999: age, method and geography

**DOI:** 10.1186/1471-2458-4-49

**Published:** 2004-10-20

**Authors:** Cameron Stark, Paddy Hopkins, Diane Gibbs, Tracey Rapson, Alan Belbin, Alistair Hay

**Affiliations:** 1 Centre for Rural Health, University of Aberdeen, The Green House, Beechwood Business Park North, Inverness, IV2 3ED, Scotland, UK; 2 NHS Highland, Inverness, Scotland, UK; 3 Information and Statistics Division, NHS Scotland, Edinburgh, Scotland, UK; 4 Health Centre, Durness, Sutherland, Scotland, UK; 5 New Craigs Hospital, Inverness, Scotland, UK

## Abstract

**Background:**

Male suicide rates continued to increase in Scotland when rates in England and Wales declined. Female rates decreased, but at a slower rate than in England and Wales. Previous work has suggested higher than average rates in some rural areas of Scotland. This paper describes trends in suicide and undetermined death in Scotland by age, gender, geographical area and method for 1981 – 1999.

**Methods:**

Deaths from suicide and undetermined cause in Scotland from 1981 – 1999 were identified using the records of the General Registrar Office. The deaths of people not resident in Scotland were excluded from the analysis. Death rates were calculated by area of residence, age group, gender, and method. Standardised Mortality Ratios (SMRs) and 95% confidence intervals were calculated for rates by geographical area.

**Results:**

Male rates of death by suicide and undetermined death increased by 35% between 1981 – 1985 and 1996 – 1999. The largest increases were in the youngest age groups. All age female rates decreased by 7% in the same period, although there were increases in younger female age groups.

The commonest methods of suicide in men were hanging, self-poisoning and car exhaust fumes. Hanging in males increased by 96.8% from 45 per million to 89 per million, compared to a 30.7% increase for self-poisoning deaths. In females, the commonest method of suicide was self-poisoning. Female hanging death rates increased in the time period.

Male SMRs for 1981 – 1999 were significantly elevated in Western Isles (SMR 138, 95% CI 112 – 171), Highland (135, CI 125 – 147), and Greater Glasgow (120, CI 115 – 125). The female SMR was significantly high only in Greater Glasgow (120, CI 112 – 128).

**Conclusion:**

All age suicide rates increased in men and decreased in women in Scotland in 1981 – 1999. Previous findings of higher than expected male rates in some rural areas were supported. Rates were also high in Greater Glasgow, one of the most deprived areas of Scotland. There were changes in the methods used, with an increase in hanging deaths in men, and a smaller increase in hanging in women. Altered choice of method may have contributed to the increased male deaths.

## Background

Compared to the adjacent countries of England and Wales, Scotland had a low suicide rate through most of the twentieth century [1]. This did not appear to be explained by differences in recording of suicide [2]. Suicide rates in Scottish men increased in the 1970s and 1980s [3,4]. Rates in younger men continued to increase in the late 1980s [5] and early 1990s [6], at a time when male rates in England declined [7,8]. By contrast, female rates decreased in Scotland, although not as rapidly as in England and Wales [3].

Several authors have noted the importance of suicide in Scotland as a public health problem [9,10]. There was an increase in hanging and motor vehicle exhaust fumes as methods of male suicide in 1970 – 1989 and Pounder [5] suggested that choice of method might contribute to the increase in Scottish male rates, as some methods are associated with higher case fatality rates. Crombie [11] found that some areas had higher rates of male suicide than the Scottish average, mainly in rural areas. Access to particular methods of suicide may contribute to this [12]. Gender, age, suicide method and geographical area therefore appear to be important considerations in the epidemiology of suicide in Scotland. No recent summary of suicide trends in Scotland has been available, and this paper describes trends in relation to these factors.

## Methods

We used anonymised information on deaths by suicide and undetermined deaths provided by the General Register Office for Scotland (GROS). Deaths registered during 1981 – 1999 were included if the cause of death was recorded as suicide or as undetermined cause (ICD-9 E950-E959 and E980-E989 respectively). Undetermined deaths were included as suicide deaths may be misattributed [13,14].

Population figures were taken from the GROS annual reports for the mid-year of each period. For analyses by area, if a death was registered away from the person's home address, the death was allocated to their area of residence, rather than the area in which they died. Deaths of people resident outside Scotland were identified using country codes, and were excluded. As far as possible, therefore, results reflect the rates of suicide and undetermined deaths of people resident in each area of Scotland. Standardised Mortality Ratios were calculated for National Health Service administrative areas, with 95% confidence intervals. In time period descriptions, the periods 1981 – 1985, 1986 – 1990, 1991 – 1995, and 1996 – 1999 were used. Data were analysed using Excel and SPSS.

## Results

There were 14502 deaths recorded as suicide or undetermined cause in the time period. Of these deaths, 28.5% occurred in females (n = 4137) and 71.5% in males (n = 10365).

### Gender and age group

Table 1 shows changes by gender. The male suicide rate for suicide and undetermined deaths increased from 187 per million in 1981 – 1985 to 252 per million in 1996 – 1999, an increase of 35%. In the same period, the female rate per million decreased from 88 to 82 per million, a 7% decrease. The female decline occurred between 1981 – 1985 and 1986 – 1999. By contrast, male rates increased between all time periods. The female: male rate ratio in 1981 – 1985 was 2.1:1 By 1996 – 1999 this had increased to 3.1:1.

**Table 1:** Suicide and Undetermined Deaths in Scotland 1981 – 1999 By Gender and Time Period Rate per Million Population

	**1981 to 1985**		**1986 to 1990**		**1991 to 1995**		**1996 to 1999**		
		
**Gender**	**No. of deaths**	**Rate/million**	**No. of deaths**	**Rate/million**	**No. of deaths**	**Rate/million**	**No. of deaths**	**Rate/million**	**% change from first to last time period**
**Males**	2324	*187*	2,587	*210*	2,948	*238*	2,506	*252*	35%
**Females**	1180	*88*	1,030	*78*	1,058	*80*	869	*82*	-7%
**Total**	3,504	*136*	3,617	*142*	4,006	*156*	3,375	*165*	21%

Examining changes by age group in males (Table 2), there are increases in male rates in the under 15 years, 15– 24 year, 25 – 34 year and 35 – 44 year age groups, of 137%, 97%, 86% and 26% respectively. The increase in the youngest male age group, although based on very small numbers of deaths, occurred between 1986 – 90 and 1991 – 95. In the 15–24 and 25 – 34 year age groups, increases occurred in every time period. There were decreases in the 45 – 54 and 55 – 64 year age groups and increases, of 4% and 10%, in the 65 – 74 and 75 years and over age groups.

**Table 2:** Suicide and Undetermined Deaths in Males in Scotland 1981 – 1999 By Age Group and Time Period

	**1981–1985**		**1986–1990**		**1991–1995**		**1996–1999**		
		
**Age group**	**No. of deaths**	**Rate per million**	**No. of deaths**	**Rate per million**	**No. of deaths**	**Rate per million**	**No. of deaths**	**Rate per million**	**% change from first to last period**
**<15 years**	11	*4.1*	11	*4.5*	25	*10.1*	19	*9.7*	137%
**15–24**	317	*141.0*	440	*208.3*	467	*257.2*	369	*278.0*	97%
**25–34**	410	*226.1*	536	*276.1*	736	*355.9*	677	*421.4*	86%
**35–44**	422	*268.5*	470	*279.2*	594	*340.8*	506	*338.0*	26%
**45–54**	437	*311.0*	420	*301.6*	473	*313.6*	377	*290.6*	-7%
**55–64**	382	*290.7*	339	*263.3*	306	*241.3*	229	*226.4*	-22%
**65–74**	228	*244.6*	223	*238.5*	212	*216.0*	200	*254.3*	4%
**75+**	117	*253.6*	148	*287.8*	135	*253.6*	129	*278.8*	10%
**All Ages**	2,324	*187.0*	2,587	*209.7*	2,948	*237.8*	2,506	*252.1*	35%

In women, there were increases in the three youngest age groups, with a 76% increase in rates in the under 15 year old group, 150% in the 15 – 24 year group and 37% in 25 – 34 year olds (Table 3). There were decreases in every older age group from 35 – 44 years to 75 years and over.

**Table 3:** Suicide and Undetermined Deaths in Females in Scotland 1981 – 1999 By Age Group and Time Period

	**1981–1985**		**1986–1990**		**1991–1995**		**1996–1999**		
		
**Age group**	**No. of deaths**	**Rate per million**	**No. of deaths**	**Rate per million**	**No. of deaths**	**Rate per million**	**No. of deaths**	**Rate per million**	**% change from first to last period**
**<15 years**	7	*2.7*	7	*3.0*	7	*3.0*	9	*4.8*	76%
**15–24**	70	*32.4*	89	*44.1*	100	*57.6*	103	*81.0*	150%
**25–34**	141	*78.9*	174	*91.4*	217	*106.4*	172	*108.3*	37%
**35–44**	179	*112.3*	158	*93.3*	192	*109.1*	157	*103.9*	-8%
**45–54**	231	*154.9*	151	*103.3*	179	*115.0*	153	*115.1*	-26%
**55–64**	264	*176.3*	187	*129.6*	138	*98.4*	96	*86.7*	-51%
**65–74**	174	*136.5*	153	*122.5*	107	*84.8*	101	*102.6*	-25%
**75+**	114	*114.3*	111	*103.0*	118	*108.3*	78	*87.0*	-24%
**All Ages**	1,180	*88.4*	1,030	*78.1*	1,058	*80.1*	869	*82.4*	-7%

### Method of suicide

The commonest methods of suicide and undetermined deaths in men were hanging, strangulation and suffocation, poisoning with solid or liquid substances, drowning, use of gases and vapours and jumping from high places (Table 4). Hanging, strangulation and suffocation in males had a similar rate to poisoning with solid or liquid substances in 1981 – 1985, but by 1996 – 1999 it had increased by 96.8% from 45 per million to 89 per million, compared to a 30.7% increase for self-poisoning deaths, from 46 per million to 60 per million. Deaths from jumping and cutting also increased, by 44.2% and 18.8% respectively. 'Other gases and vapours', predominantly car exhaust deaths (data not presented), decreased slightly from the first to last periods, but this concealed a substantial increase between 1981 – 1985 and 1986 – 1990, followed by a decrease in 1996 – 1999. Unspecified means increased by 70.9% from 14 to 23 per million.

**Table 4:** Methods of Suicide and Undetermined Death in Males in Scotland 1981 – 1999 By Time Period

	**1981 to 1985**		**1986 to 1990**		**1991 to 1995**		**1996 to 1999**		
		
**Primary Cause**	**No. of Deaths**	**Rate/million**	**No. of Deaths**	**Rate/million**	**No. of Deaths**	**Rate/million**	**No. of Deaths**	**Rate/million**	**% change from first to last time period**
Solid or liquid substances	572	*46*	594	*48*	768	*62*	598	*60*	30.7
Hanging, strangulation and suffocation	562	*45*	630	*51*	774	*62*	880	*89*	96.8
Submersion(drowning)	353	*28*	417	*34*	358	*29*	268	*27*	-5.1
Other gases and vapours	288	*23*	428	*35*	448	*36*	225	*23*	-2.3
Other, unspecified means	169	*14*	170	*14*	226	*18*	231	*23*	70.9
Firearms and explosives	166	*13*	119	*10*	114	*9*	66	*7*	-50.3
Jumping from high place	163	*13*	176	*14*	203	*16*	188	*19*	44.2
Cutting and piercing instruments	40	*3*	38	*3*	38	*3*	38	*4*	18.8
Gases in domestic use	8	*1*	7	*1*	7	*1*	8	*1*	33.4
Late effects of injury	3	*. 4*	8	*1*	12	*1*	4	*1*	99.9
Total	2,324	*187*	2,587	*210*	2,948	*238*	2,506	*252*	34.8

In females, the commonest methods of suicide and undetermined death were poisoning with solid or liquid substances, hanging, strangulation and suffocation, and drowning (Table 5). Self-poisoning decreased by 4.8% between first and last periods from 45 to 43 per million, while drowning, and jumping from high places decreased by 54.1% and 30.3% respectively. Gases and vapours showed an increase but, as in males, the rate was higher in the middle two time periods. The rate of hanging, strangulation and suffocation deaths increased by 53.5%. There was an increase in unspecified means of suicide, from 4 to 8 per million.

**Table 5:** Methods of Suicide and Undetermined Death in Females in Scotland 1981 – 1999 By Time Period

	**1981 to 1985**		**1986 to 1990**		**1991 to 1995**		**1996 to 1999**		
		
**Primary Cause**	**No. of Deaths**	**Rate/million**	**No. of Deaths**	**Rate/million**	**No. of Deaths**	**Rate/million**	**No. of Deaths**	**Rate/million**	**% change from first to last time period**
Solid or liquid substances	601	*45*	577	*44*	570	*43*	452	*43*	-4.8
Submersion(drowning)	248	*19*	159	*12*	132	*10*	90	*9*	-54.1
Hanging, strangulation and suffocation	127	*10*	95	*7*	134	*10*	154	*15*	53.5
Jumping from high place	89	*7*	77	*6*	67	*5*	49	*5*	-30.3
Other, unspecified means	60	*4*	53	*4*	81	*6*	83	*8*	75.1
Other gases and vapours	31	*2*	51	*4*	50	*4*	30	*3*	22.5
Firearms and explosives	13	*1*	10	*1*	6	*0.5*	3	*0.4*	-68.9
Cutting and piercing instruments	9	*1*	8	*1*	9	*1*	5	*1*	-25.0
Late effects of injury	2	*0.4*	-	*-*	7	*1*	3	*0.4*	1.4
Gases in domestic use	-	*-*	-	*-*	2	*0.4*	-	*-*	-
Total	1,180	*88*	1,030	*78*	1,058	*80*	869	*82*	-6.8

### Geographical areas

**Figure 1:**
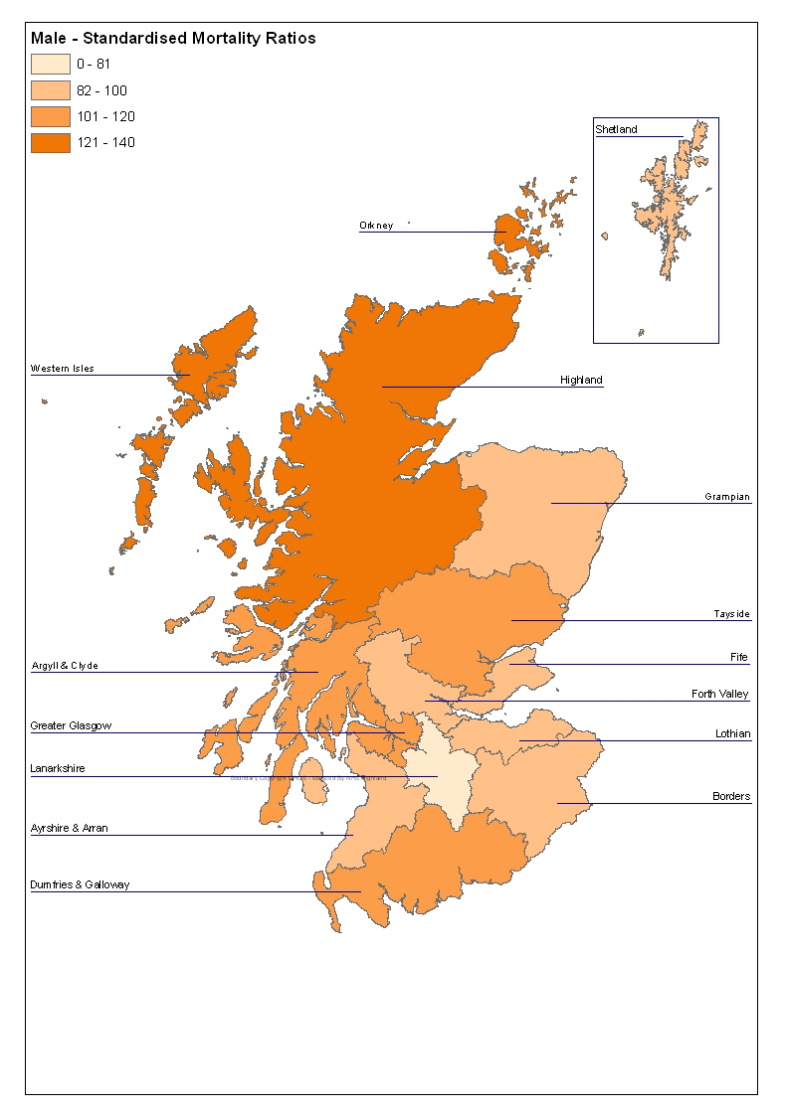
Male Standardised Mortality Ratios for Suicide and Undetermined Deaths in Scotland, 1981 – 1999 by Area

**Figure 2:**
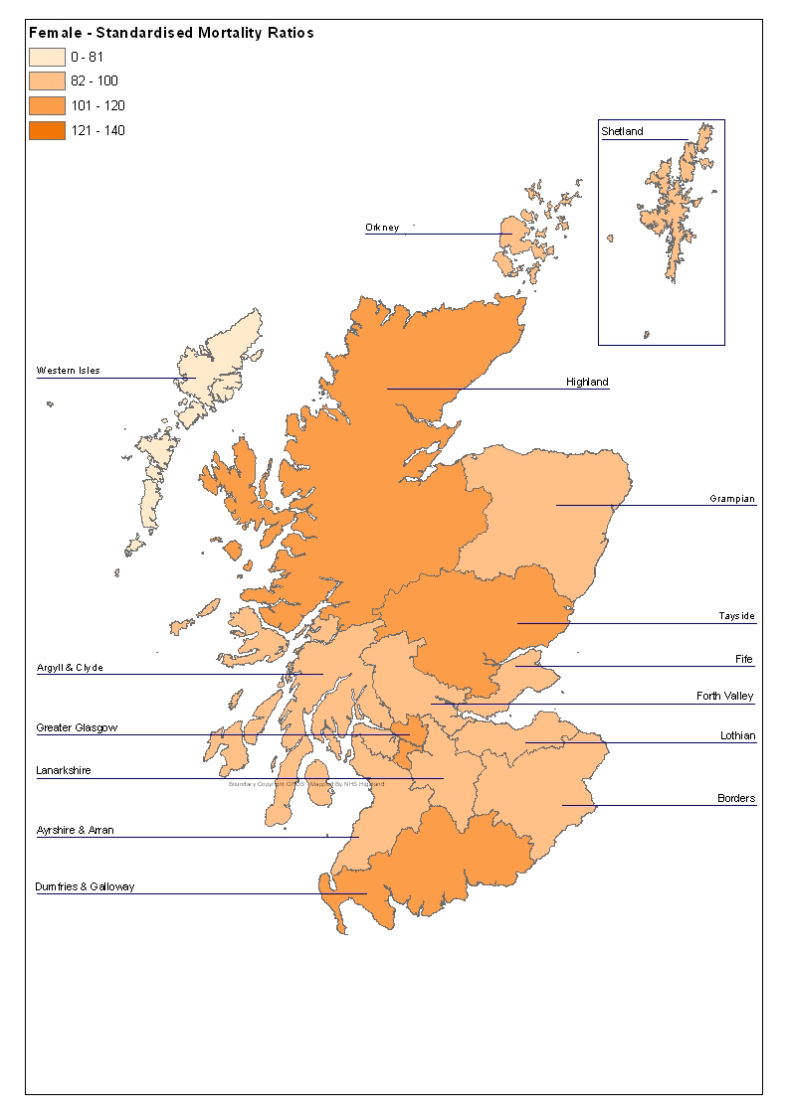
Female Standardised Mortality Ratios for Suicide and Undetermined Deaths in Scotland, 1981 – 1999 by Area

In the nineteen-year period as a whole, there was substantial geographical variation (Figures 1 and 2). The highest male rates were in Western Isles, Highland, Orkney, Greater Glasgow and Tayside (Table 6). When considered as Standardised Mortality Ratios, Western Isles, Highland and Greater Glasgow were statistically significantly elevated (Table 6). Six areas, Fife, Ayrshire and Arran, Forth Valley, Lothian, Borders and Lanarkshire had significantly lower SMRs than the Scottish average.

**Table 6:** Male Standardised Mortality Ratios by Health Service Area Suicide and Death by Undetermined Cause in Scotland 1981 – 1999

**Health Board of Residence**	**No. of Deaths**	**Rate/million**	**Standardised Mortality Ratio**		
			
			**Ratio**	**LCI**	**UCI**
Argyll & Clyde	909	225	103	97	110
Ayrshire & Arran	676	197	91	84	98
Borders	176	186	84	72	97
Dumfries & Galloway	301	223	101	90	113
Fife	634	197	90	83	97
Forth Valley	496	197	89	82	98
Grampian	1,050	219	99	93	105
Greater Glasgow	2,252	264	120	115	125
Highland	555	294	135	125	147
Lanarkshire	907	174	81	75	86
Lothian	1,399	202	90	85	95
Orkney	50	274	124	92	164
Shetland	47	214	99	72	133
Tayside	828	231	105	98	112
Western Isles	85	300	138	112	171
Scotland	10,365	220	100	-	-

In women, the highest rates were in Glasgow, Tayside, Highland and Dumfries and Galloway (Table 7). Only the Greater Glasgow SMR was significantly elevated. One area, Lanarkshire, had a significantly low female SMR.

**Table 7:** Female Standardised Mortality Ratios by Health Service Area Suicide and Death by Undetermined Cause in Scotland 1981 – 1999

**Health Board of Residence**	**No. of Deaths**	**Rate/million**	**Standardised Mortality Ratio**		
			
			**Ratio**	**LCI**	**UCI**
Argyll & Clyde	330	77	93	84	104
Ayrshire & Arran	290	78	95	84	106
Borders	83	81	95	77	118
Dumfries & Galloway	122	85	101	84	120
Fife	277	82	100	89	112
Forth Valley	195	73	89	77	102
Grampian	383	77	95	86	105
Greater Glasgow	921	99	120	112	128
Highland	169	86	105	91	123
Lanarkshire	366	66	83	75	92
Lothian	598	81	97	90	105
Orkney	15	80	98	55	162
Shetland	16	74	95	54	154
Tayside	357	92	110	99	122
Western Isles	15	53	65	36	107
Scotland	4,137	82	100	-	-

There were changes within areas over the period studied. Male rates increased in all fifteen NHS Board areas (Table 8). The smallest percentage increases were in Orkney, Highland and Greater Glasgow, three of the areas with the highest male rates in the first time period. Shetland and Western Isles had large percentage increases, but this was based on small numbers of suicide and undetermined cause deaths. The largest increases in mainland Scottish Board areas were in Argyll and Clyde (62%), Borders (60%), Forth Valley 59%), Ayrshire and Arran (56%) and Grampian (49%).

**Table 8:** Male Deaths from Suicide and Undetermined Cause in Scotland 1981 – 1999 Rates By Health Service Area and Time Period

	**1981 to 1985**		**1986 to 1990**		**1991 to 1995**		**1996 to 1999**		
		
**Primary Cause**	**No. of Deaths**	**Rate/million**	**No. of Deaths**	**Rate/million**	**No. of Deaths**	**Rate/million**	**No. of Deaths**	**Rate/million**	**% change from first to last time period**
Argyll & Clyde	190	*175*	212	*199*	272	*259*	235	*283*	62
Ayrshire & Arran	143	*158*	161	*178*	194	*214*	178	*246*	56
Borders	31	*128*	41	*167*	62	*245*	42	*205*	60
Dumfries & Galloway	71	*201*	74	*209*	79	*220*	77	*269*	34
Fife	148	*177*	155	*183*	169	*198*	162	*239*	35
Forth Valley	102	*154*	127	*192*	136	*205*	131	*245*	59
Grampian	219	*181*	258	*208*	291	*224*	282	*270*	49
Greater Glasgow	551	*234*	565	*252*	666	*304*	470	*270*	15
Highland	131	*273*	155	*315*	144	*284*	125	*305*	12
Lanarkshire	195	*140*	242	*177*	255	*187*	215	*197*	40
Lothian	307	*172*	350	*195*	408	*223*	334	*222*	29
Orkney	13	*275*	11	*233*	15	*308*	11	*281*	2
Shetland	12	*202*	10	*179*	7	*121*	18	*388*	92
Tayside	193	*205*	200	*214*	230	*242*	205	*272*	33
Western Isles	18	*230*	26	*343*	20	*274*	21	*376*	64
Scotland	2,324	*187*	2,587	*210*	2,948	*238*	2,506	*252*	35

In females, rates changed little or decreased in all mainland Boards other than Argyll and Clyde (16.9% increase) (Table 9). Rates increased in Orkney, Shetland and Western Isles, although these were based on very small numbers of deaths. The greatest declines in mainland Board areas were in Ayrshire and Arran (30% decrease) and Grampian (27.9% decrease).

**Table 9:** Female Deaths from Suicide and Undetermined Cause in Scotland 1981 – 1999 Rates By Health Service Area and Time Period

	**1981 to 1985**		**1986 to 1990**		**1991 to 1995**		**1996 to 1999**		
		
**Primary Cause**	**No. of Deaths**	**Rate/million**	**No. of Deaths**	**Rate/million**	**No. of Deaths**	**Rate/million**	**No. of Deaths**	**Rate/million**	**% change from first to last time period**
Argyll & Clyde	96	*82*	73	*64*	76	*68*	85	*96*	17
Ayrshire & Arran	88	*90*	73	*75*	80	*82*	49	*63*	-30
Borders	26	*98*	20	*75*	18	*66*	19	*86*	-12
Dumfries & Galloway	34	*91*	29	*77*	32	*84*	27	*89*	-2
Fife	79	*89*	76	*85*	69	*77*	53	*74*	-18
Forth Valley	53	*75*	44	*63*	55	*78*	43	*76*	0.3
Grampian	133	*105*	85	*66*	84	*63*	81	*76*	-28
Greater Glasgow	270	*104*	238	*97*	235	*98*	178	*94*	-10
Highland	41	*82*	40	*79*	59	*112*	29	*68*	-17
Lanarkshire	98	*67*	93	*64*	93	*64*	82	*71*	7
Lothian	165	*85*	156	*81*	143	*73*	134	*84*	-0.3
Orkney	2	*41*	2	*41*	3	*60*	8	*202*	390
Shetland	1	*17*	4	*72*	7	*125*	4	*88*	412
Tayside	91	*88*	93	*92*	101	*99*	72	*89*	0.6
Western Isles	3	*38*	4	*53*	3	*41*	5	*88*	130
Scotland	1,180	*88*	1,030	*78*	1,058	*80*	869	*82*	-7

## Discussion

The epidemiology of suicide in Scotland has changed greatly between 1981 and 1999. Male suicide rates have increased in all age groups up to and including 35 – 44 years. The highest male suicide and undetermined death rates in 1996 – 1999 were in the 25 – 34 year age group. In women, rates dropped in age groups from 35 – 44 years up to and including 75 years and over. Rates increased in younger women.

There is limited information on the factors underlying individual deaths from suicide in Scotland. Squires and Gorman [15] reviewed the deaths by suicide of a group of young men in Lothian, and reported that a third had experienced recent relationship difficulties with a partner. Half of the group studied had a previous history of attempted suicide. Cavanagh et al [16] reported largely similar findings in a case control study in south-east Scotland. The group who had died by suicide had an odds ratio of 9.0 (95% CI 1.3 – 399) for current family problems, and an odds ratio of 5.0 (95% CI 1.1 – 47) for physical health problems. There was felt to be limited scope to intervene in suicide and deliberate self-harm through family health services because of limited contact, and non-specific presentation of problems [15,17].

Some methods of self-harm have higher case fatality rates [18]. Firearms have the highest case fatality rates, followed by drowning and hanging [19,20]. The most striking changes in male suicide methods in Scotland were the marked increase in hanging deaths, and the increase and subsequent decrease in deaths from 'other gases and vapours', which are mainly car exhausts. It seems likely that the decrease in motor vehicle exhaust fume deaths was related to the introduction of catalytic converters. Not all countries have reported a decrease in suicide from motor vehicle exhausts after catalytic converter introduction [21], but deaths in England decreased [22]. The reduction in deaths from motor vehicle exhaust fumes in England and Wales was associated with an increase in hanging deaths [7]. In Scotland, our data suggest that hanging deaths were increasing in men before deaths from motor vehicle exhaust fumes began to decline. The increase in hanging also appears greater than the decrease in motor vehicle exhaust deaths. The relationship between vehicle exhaust fume and hanging deaths in Scotland does not appear to be identical to that reported in England and Wales, and deserves further investigation.

The difference between areas was also of note. The lower rates of increase in the areas with the highest initial rates may reflect to regression to the mean. Method availability [23] may be important in rural/urban differences. Obafunwa and Busuttil [24] reported that, within the Lothian region of Scotland, hanging was commoner in younger deaths, while use of car exhaust fumes for suicide was particularly important in rural areas [24,25]. In Lothian, an area that includes the capital city of Scotland, suicide by firearms was uncommon. Previous work has suggested higher rates of male suicide in some rural parts of Scotland [11]. Stark et al [12] have suggested that this may be related to the use of methods of self-harm in rural areas, such as firearms, with a high case fatality rate. Gunnell and colleagues [26] have argued, in relation to England and Wales, that changes in method preference, and therefore in case fatality, should be considered before concluding that changes must relate to social trends.

Availability of method would not explain the differences between apparently similar rural areas. Previous work has found that deprived areas of Scotland tend to have higher suicide rates [27,28]. Deprived areas in Scotland were reported to have had the greatest increase in young male suicide between 1981 – 3 and 1991 – 3 [29]. Greater Glasgow, the non-rural area with the highest rate over the time period, is an area with substantial deprivation. Rural deprivation is difficult to measure, and recent work suggests that rural areas of Scotland may suffer greater levels of deprivation than had been realised. It is possible that rural deprivation is underestimated, and deprivation may explain more of the elevation in some rural areas than has been assumed in the past.

Using routine information allowed a large number of suicide and undetermined deaths to be included in this series. There are, however, limitations to the use of anonymised routine data. No qualitative information was available, and our exploration of the data was limited to trends with no examination of possible underlying causes. The increase in the rate of deaths recorded as suicide or undetermined cause of death, but where no detail on method was included, could conceal recent trends. The increase as a percentage of relevant registrations was small, however, increasing from 7.5% in the first period to 9.1% in the last period studied. The classification of deaths as suicide is often difficult, but the inclusion of undetermined deaths as well as deaths recorded as suicide should have helped to minimise bias from under identification [14,30]. Squires et al [31] reported that improved communication between pathologists and the Registrar General for Scotland from 1994 on was associated with a decrease in undetermined deaths and in increase in deaths coded as being caused by dependent or non-dependent use of drugs. It is possible, therefore, that the figures for the final two periods may under-represent deaths that would have been identified as 'unidentified' in the earlier periods. Using information on Scottish residents only allowed identification of the suicide rates of local populations. Deaths of non-residents can account for up to 10% of all suicide and undetermined cause deaths in some rural areas of Scotland [12]. Our findings indicate that, even when these deaths are excluded, rates remain increased in some rural areas.

## Conclusions

A divergence between male rates in England and Wales and in Scotland, and in male and female rates within Scotland, had been identified for the first part of the time period described here. This work found that male rates of suicide and undetermined death continued to increase in Scotland, but also identifies increases in younger female age groups. Examination of changes in method by male and female age group will help to establish whether changes in case fatality because of altered method choice [26] may be part of the explanation for these findings. The shift to hanging seems to be a significant trend in men in Scotland. It will be important to understand the reasons for this to allow appropriate intervention strategies to be considered.

Some rural areas of Scotland had significantly elevated male suicide rates. We have suggested that access to lethal means of suicide may be one contributing mechanism for this, and have also noted the higher than expected suicide numbers in some rural occupations [12]. Rural areas are subject to poverty of income and opportunity, so it is also possible that rural deprivation may play an important part. Occupational associations of suicide in Scotland deserve further exploration. Examination of the association between deprivation, rurality and suicide may assist in the identification of possible interventions in rural Scotland.

## Competing interests

The authors declare that they have no competing interests.

## Authors' contributions

CS had the idea for the study, wrote the grant application, contributed to the design and interpretation and drafted the paper. Diane Gibbs and Tracey Rapson analysed the data. Paddy Hopkins contributed to the design, undertook part of the analysis, and commented on the interpretation of the results. Alan Belbin and Alistair Hay contributed to the design and helped interpret the results. All authors read and approved the final draft of the paper.

## Pre-publication history

The pre-publication history for this paper can be accessed here:


